# Supplementary figures and images for: Mendelian segregation and high recombination rates facilitate genetic analyses in Cryptosporidium parvum
Source: PLoS Genet. 2024 Jun 17;20(6):e1011162. doi: 10.1371/journal.pgen.1011162 (PMC11213348; doi:10.1371/journal.pgen.1011162)

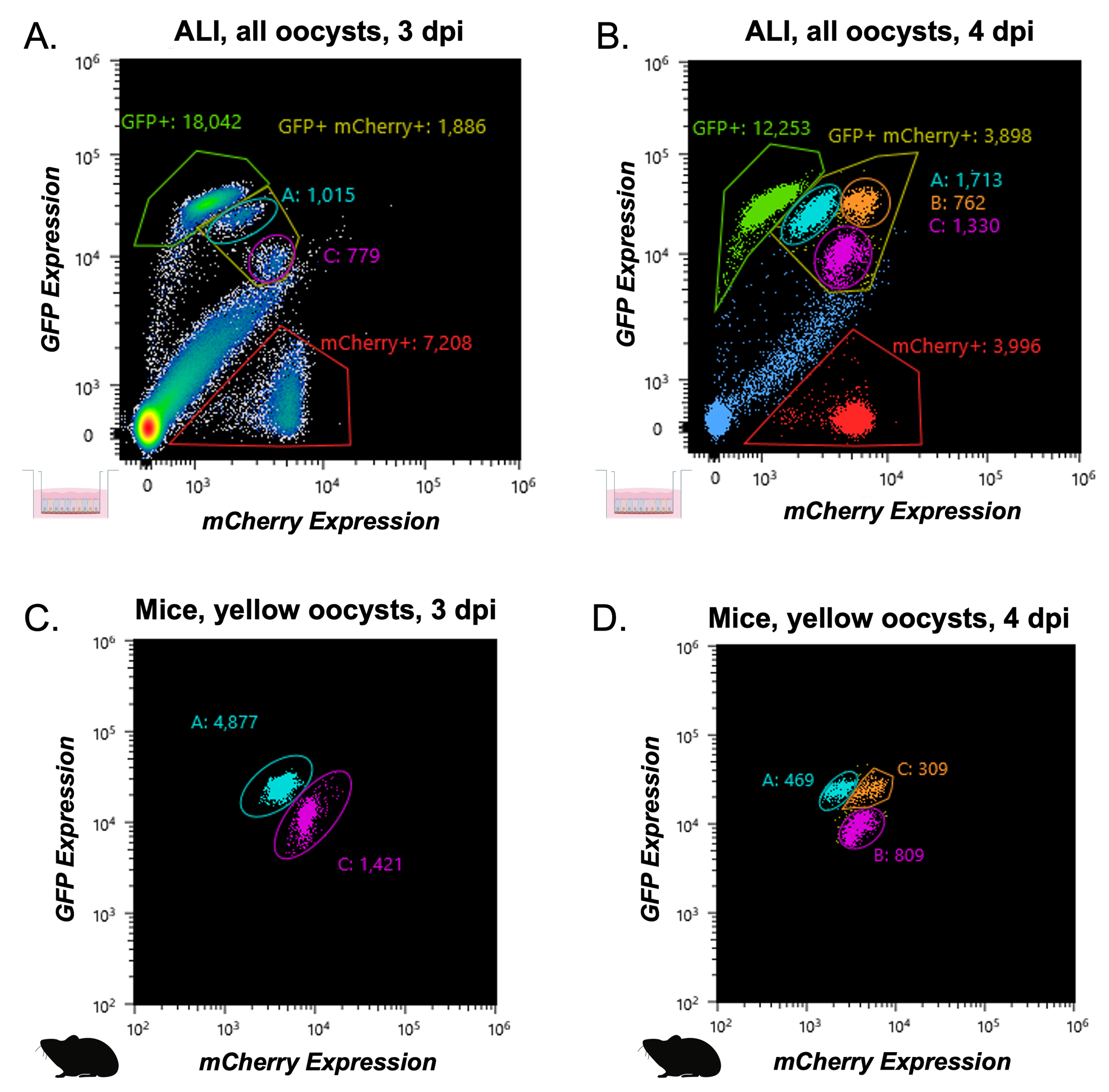

Supplement: S1 Fig — (A) ALI was infected with Δuprt-mCherry and Δtk-GFP oocysts, after 3 days post infection (dpi) transwells were scraped, bleached, and the expression of F1 oocysts were examined by flow cytometry. The Δtk-GFP oocysts produced by self-fertilization are more frequent than Δuprt-mCherry oocysts or outcrossed “yellow” oocysts. Yellow oocysts (mCherry+/GFP+) have two different populations that vary slightly in expression. GFPhi/mCherrymid and GFPmid/mCherryhi yellow oocysts occur at similar rates and likely differ based on the genotype of macrogamont”mother” of the oocyst. (B) A third population of mCherry+/GFP+ oocysts (GFPhi/mCherryhi) can be found in ALI cultures harvested at 4 days post infection using methods described above. These oocysts are likely the result of F1 yellow macrogamonts mating with green and red microgamonts and are indicative that a second round of meiosis has begun. (C) The GFPhi/mCherrymid and GFPmid/mCherryhi yellow oocyst populations can also be found in samples collected from Ifngr1−/− mice infected with Δuprt-mCherry and Δtk-mGFP harvested after 3 days of infection. (D) Similarly, a third population of GFPhi /mCherryhi yellow oocysts can be found in samples collected from mice at 4 days post infection using methods described above. To capture only F1 oocysts all experiments utilized only oocysts harvested at 3 dpi. (TIF) [file pgen.1011162.s001.tif]

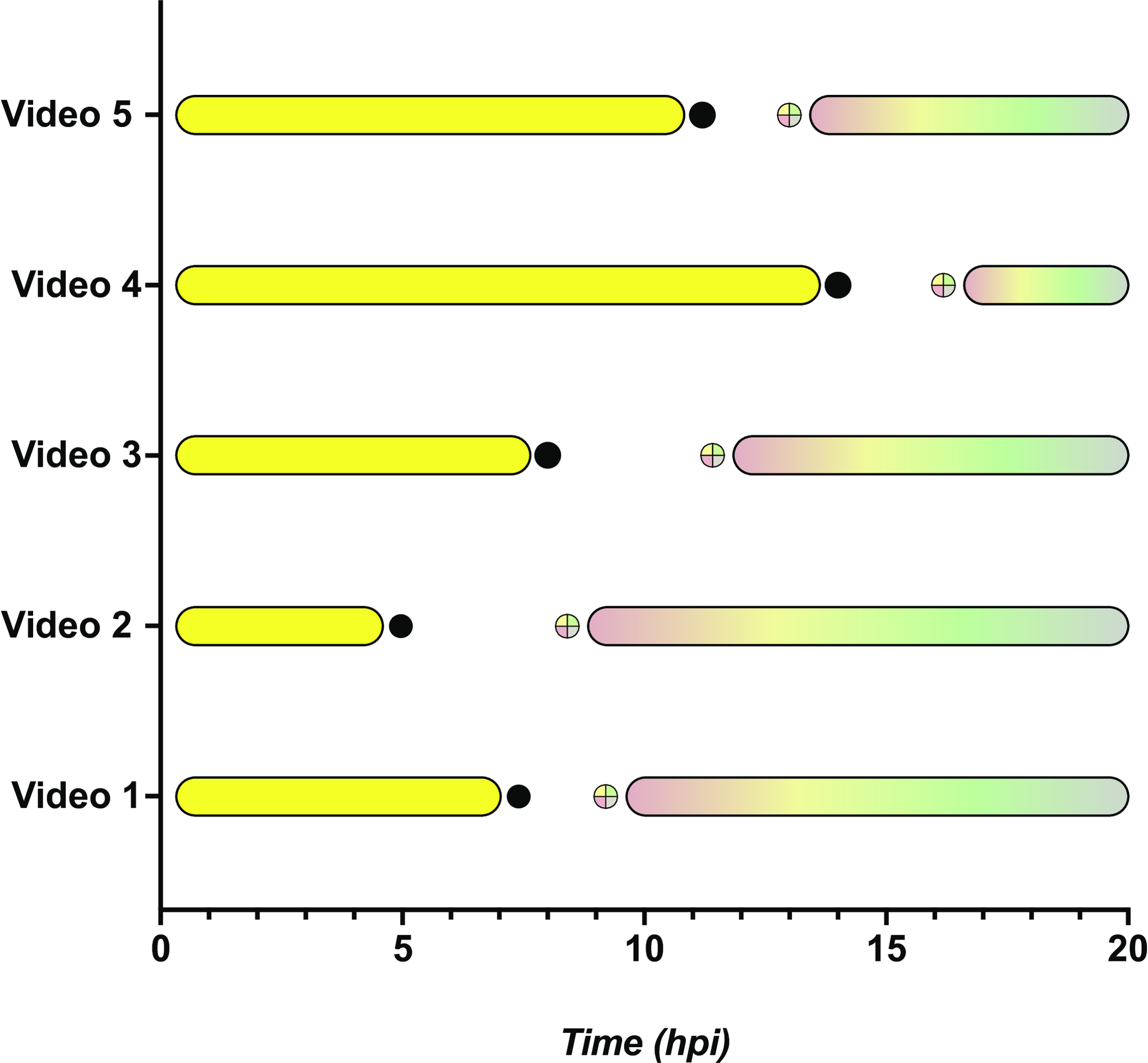

Supplement: S2 Fig — Yellow bars represent the time (hours post infection) where parentally inherited cytosolic fluorescent proteins were expressed in F1 ‘yellow’ progeny and black dots indicate when these parentally inherited proteins were degraded. The colored dot represents the time when the germline encoded fluorescent protein began to be expressed and were continuously expressed throughout the first phase of merogony. The progeny from an individual F1 “yellow” oocyst were analyzed across 5 videos in two independent experiments. Each row represents progeny from an individual F1 “yellow” oocyst (n = 5) across two independent experiments (S1 Dataset). (TIF) [file pgen.1011162.s002.tif]

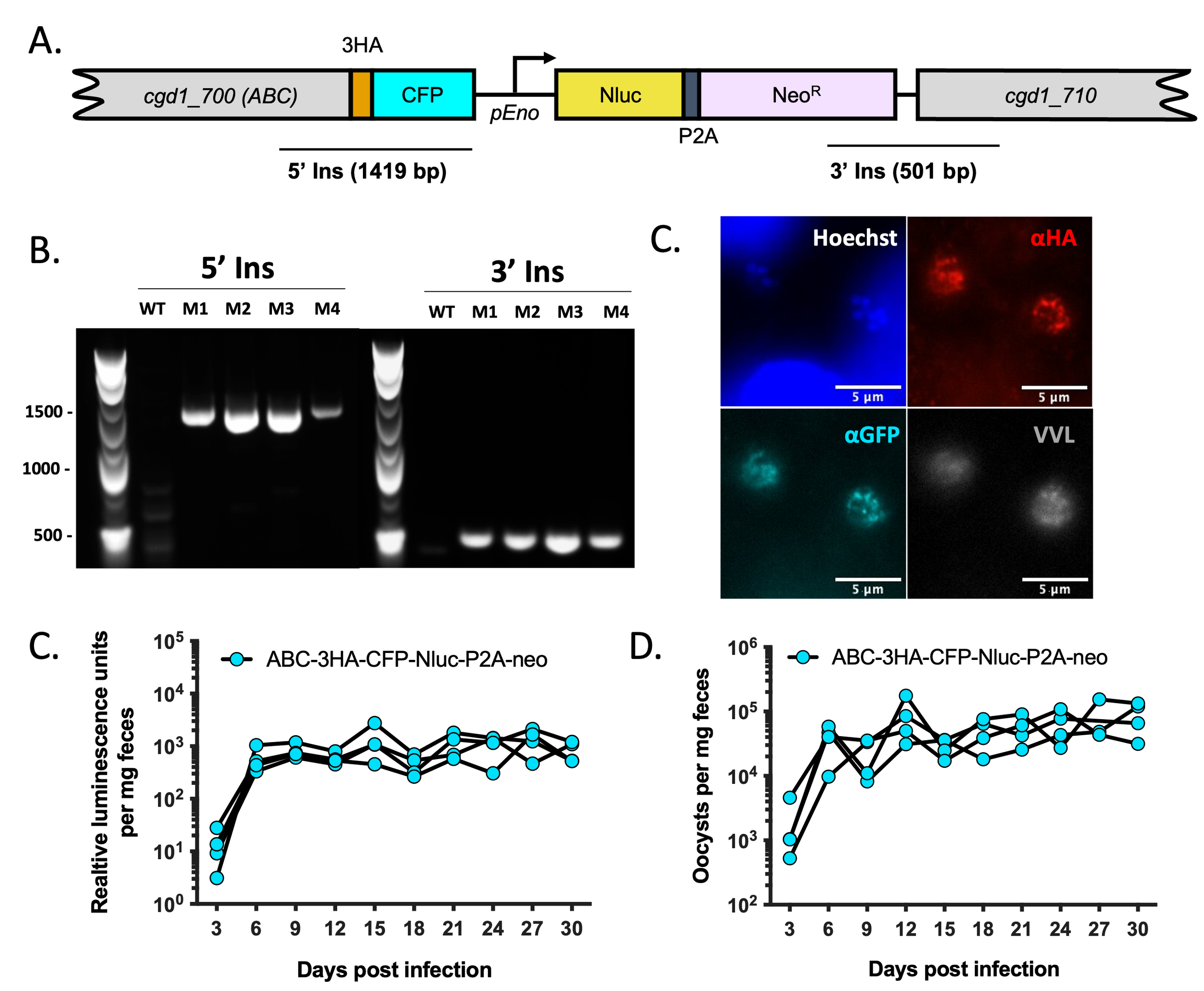

Supplement: S3 Fig — (A) A diagram of the ABC-3HA-CFP targeting vector and the expected sizes for the 5’ Ins (1419 bp) and 3’ Ins (501 bp) amplicons used for the confirmation of proper insertion by PCR. (B) PCR analysis of ABC-3HA-CFP oocysts amplified in four NSG mice. WT, wild type. M1-4, DNA purified from fecal samples collected from each mouse used for amplification. The 5′ Ins and 3’ Ins products are specific for the 5′ and 3’ insertion sites of the integrated construct. Primers are identified in S2 Table. (C) ABC-HA-CFP oocysts purified from mouse feces were used for infection of HCT-8 cells, fixed at 18 hpi and stained with rabbit anti-GFP (also recognizes CFP), rat anti-HA, and VVL-Biotin all at 1:500. Followed by a secondary stain of Alexa Fluor 488 goat anti-rabbit IgG, Alexa Fluor 568 goat anti-rat IgG, and Alexa Fluor 647 Streptavidin all at a 1:1000 dilution. Hoechst was used for nuclear staining at 1:2000. (C) Fecal pellets collected from NSG mice from 3 to 30 days post infection were used for nanoluciferase assays and expressed as relative luminescence units (RLU) per milligram of feces. Each point represents a single sample from an individual mouse (n = 4). (D) DNA purified from fecal pellets collected during amplification were used for a qPCR analysis and expressed as gDNA equivalents per milligram of feces. Each point represents a single sample from an individual mouse (n = 4). (TIF) [file pgen.1011162.s003.tif]

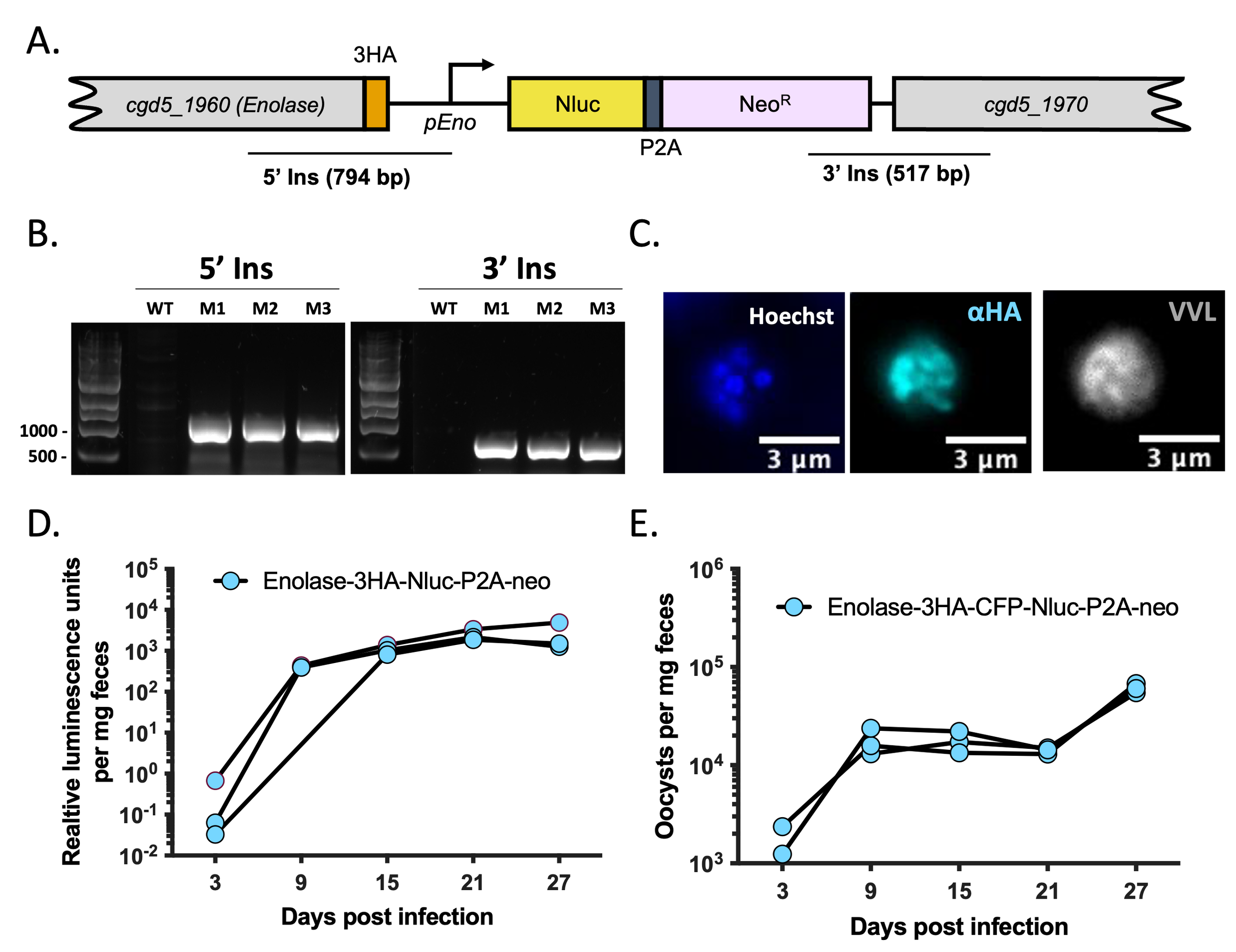

Supplement: S4 Fig — (A) A diagram of the Enolase-3HA targeting vector and the expected sizes for the 5’ Ins (749 bp) and 3’ Ins (517 bp) amplicons used for the confirmation of proper insertion by PCR. (B) PCR analysis of Enolase-3HA oocysts amplified in three NSG mice. WT, wild type. M1-3, DNA purified from fecal samples collected from each mouse used for amplification. The 5′ Ins and 3’ Ins products are specific for the 5′ and 3’ insertion sites of the integrated construct. Primers are identified in S2 Table. (C) Enolase-HA oocysts purified from mouse feces were used for infection of HCT-8 cells, fixed at 18 hpi and stained with rat anti-HA at 1:500 and VVL-Biotin at 1:500. Followed by a secondary stain of Alexa Fluor 488 goat anti-rat and Alexa Fluor 647 Streptavidin at a 1:1000 dilution. Hoechst was used to stain nuclei at 1:2000. (C) Fecal pellets collected from NSG mice from 3 to 27 days post infection were used for nanoluciferase assays and expressed as relative luminescence units (RLU) per milligram of feces. Each point represents a single sample from an individual mouse (n = 3). (D) DNA purified from fecal pellets collected during amplification were used for a qPCR analysis and expressed as gDNA equivalents per milligram of feces. Each point represents a single sample from an individual mouse (n = 3). (TIF) [file pgen.1011162.s004.tif]
