# Supplementary material for: Mendelian segregation and high recombination rates facilitate genetic analyses in Cryptosporidium parvum
Source: PLoS Genet. 2024 Jun 17;20(6):e1011162. doi: 10.1371/journal.pgen.1011162 (PMC11213348; doi:10.1371/journal.pgen.1011162)
Supplement: S1 Table — (DOCX) [file pgen.1011162.s006.docx]

**S1 Table.** Key Resources used in the experiments.

| **REAGENT or RESOURCE** | **SOURCE** | **IDENTIFIER** |
| --- | --- | --- |
| **Antibodies** | | |
| Rabbit polyclonal anti-GFP | Thermo Fisher Scientific | Cat# A11122 |
| Rat monoclonal anti-mCherry (16D7) | Thermo Fisher Scientific | Cat# M11217 |
| Rabbit monoclonal anti-HA (C29F4) | Cell Signaling Technology | Cat# 3724S |
| Mouse monoclonal 1E12 | [1] | N/A |
| *Vicia Villosa* Lectin (VVL), Biotinylated | Vector Labs | Cat# B-1235-2 |
| Alexa Fluor 488 goat anti-rat IgG (H+L) | Thermo Fisher Scientific | Cat# A11006 |
| Alexa Fluor 488 goat anti-rabbit IgG (H+L) | Thermo Fisher Scientific | Cat# A11034 |
| Alexa Fluor 568 goat anti-rat IgG (H+L) | Thermo Fisher Scientific | Cat# A11077 |
| Alexa Fluor 568 goat anti-rabbit IgG (H+L) | Thermo Fisher Scientific | Cat# A11011 |
| Alexa Fluor 647 goat anti-mouse IgG (H+L) | Thermo Fisher Scientific | Cat# A21235 |
| Alexa Fluor 647 Streptavidin | Thermo Fisher Scientific | Cat# S21374 |
| Hoechst | Thermo Fischer Scientific | Cat# C10637 |
| ***Cryptosporidium* & Bacterial Strains** | | |
| *Cryptosporidium parvum* AUCP-1 isolate | Laboratory of William Witola | N/A |
| NEB 5-alpha Competent E. coli (High Efficiency) | New England Biolabs | Cat# C2987H |
| **Chemicals, Reagents & Supplies** | | |
| Sodium taurocholate hydrate | Sigma-Aldrich | Cat# 86339 |
| Sodium chloride | Sigma-Aldrich | Cat# S7653-250G |
| Tween-20 | Sigma-Aldrich | Cat# 11332465001 |
| Y-27632 dihydrochloride ROCK inhibitor | Tocris Biosciences | Cat# 1254 |
| Fetal bovine serum | Gibco | Cat# 10-082-147 |
| Paromomycin sulfate salt | Sigma-Aldrich | Cat# P9297 |
| Bovine serum albumin | Sigma-Aldrich | Cat# A7030 |
| Poly-L-Lysine solution (0.01%) | Sigma-Aldrich | Cat# P4707 |
| Triton X-100 | Thermo Fisher Scientific | Cat# BP151 |
| ProLong Glass Antifade Mountant | Thermo Fisher Scientific | Cat# P36984 |
| Formaldehyde (methanol-free), Ultrapure EM Grade | Polysciences, Inc. | Cat# 04018-1 |
| BD Matrigel Basement Membrane Matrix | BD Biosciences | Cat# 356234 |
| GelRed nucleic acid gel stain | Biotium | Cat# 41003-1 |
| Sodium bicarbonate | ATCC | Cat# 30-2002 |
| Carbenicillin disodium salt | Sigma-Aldrich | Cat# C3416 |
| Automatic Setup Beads Kit | SONY | Product# LE-B3001 |
| 24-well glass bottom plates | Cellvis | Product# P24-0-N |
| Transwells, polyester membrane, 0.4 mm pore | Corning Costar | Product# 3470 |
| Fetal bovine serum (FBS) | Sigma-Aldrich | Cat# F6178 |
| RPMI 1640 ATCC Modification medium | Thermo Fisher Scientific | Cat# A14091-01 |
| RPMI 1640 Medium, no phenol red | Thermo Fisher Scientific | Cat# 11835030 |
| 50% Conditioned Medium | N/A | See: Wilke et al, 2020 |
| Dulbecco’s Phosphate-Buffered Saline (DPBS) | Invitrogen | Cat# 15575020 |
| Falcon 15 mL/50 mL Polystyrene Centrifuge Tubes | Corning Costar | Cat#: 352099, 430290 |
| Sterile cell strainers, 40 µm | Fisher | Cat# 22363547 |
| **Commercial Assays** | | |
| e-Myco plus Mycoplasma PCR detection kit | Boca Scientific | Cat#: 25237 |
| QIAamp DNA Mini kit | QIAGEN | Cat# 51306 |
| QIAamp Powerfecal Pro DNA kit | QIAGEN | Cat# 51804 |
| TB Green Advantage qPCR premix | Takara Bio | Cat# 639676 |
| Gibson Assembly Cloning kit | New England Biosciences | Cat# E5510S |
| Q5 Site-directed Mutagenesis kit | New England Biosciences | Cat# E0554S |
| Q5 Hot Start High-Fidelity 2X master mix | New England Biosciences | Cat# M0494S |
| SF Cell Line 4D-Nucleofector X Kit L | Lonza | Cat# V4XC-2024 |
| Nano-Glo Luciferase Assay kit | Promega | Cat# N1120 |
| **Experimental Models: Cell Lines** | | |
| Mouse: NIH/3T3 | ATCC | CRL-1658 |
| Mouse: Ileal epithelial stem cells from C57BL/6 | Laboratory of Thaddeus Stappenbeck | N/A |
| Human: HCT-8 | ATCC | CCL-244 |
| **Experimental Models: Organisms/Strains** | | |
| Mouse: Ifngr1-/- (C57BL/6 background) | Jackson Laboratories | Cat# 003288 |
| Mouse: Nod scid gamma (NSG) | Jackson Laboratories | Cat# 005557 |
| **Oligonucleotides** | | |
| Primer: *C. parvum* GAPDH forward: CGGATGGCCATACCTGTGAG | [2] | N/A |
| Primer: *C. parvum* GAPDH reverse: GAAGATGCGCTGGGAACAAC | [2] | N/A |
| **Recombinant DNA** | | |
| Plasmid: TK-GFP-Nluc-P2A-neo-TK | [2] | #134896 |
| Plasmid: UPRT-mCh-Nluc-P2A-neo-UPRT | [2] | #135015 |
| Plasmid: pABC-3HA-CFP-Nluc-P2A-neo | This paper | N/A |
| Plasmid: Enolase-3HA-Nluc-P2A-neo | This paper | N/A |
| Plasmid: pACT1:Cas9-GFP, U6:sgTK | [2] | Cat# 122852 |
| Plasmid: pACT1:Cas9-GFP, U6:sgUPRT | [2] | Cat# 122853 |
| Plasmid: pACT1:Cas9, U6:sgABC | This paper | N/A |
| Plasmid: pACT1:Cas9, U6:sgEnolase | This paper | N/A |
| **Instruments, Software, and Algorithms** | | |
| GraphPad Prism 10 | GraphPad Software | <https://www.graphpad.com/> |
| QuantStudio Design & Analysis Software | Thermo Fisher Scientific | <https://www.thermofisher.com/us/en/home/global/forms/life-science/quantstudio-3-5-software.html> |
| FIJI (ImageJ) |  | <https://fiji.sc/> |
| SH800 SONY Cell Sorter | SONY | <https://www.sonybiotechnology.com/us/instruments/sh800s-cell-sorter/> |
| Zeiss Observer Z1 inverted microscope with a Colibri LED illumination for multi-color epifluorescence | Zeiss | <https://www.zeiss.com/microscopy> |
| Hamamatsu ORCA-ER CCD Camera | Hamamatsu | <https://www.hamamatsu.com/us/en/product/cameras> |
| ZEN 2.5 software | Zeiss | <https://www.zeiss.com/microscopy/en/products/software/zeiss-zen> |
| SONY SH800S Cell Sorter software | SONY | <https://www.sonybiotechnology.com/us/instruments/sh800s-cell-sorter/software/> |
| Zeiss Axioskop Mot Plus fluorescence microscope | Zeiss | <https://www.zeiss.com/microscopy> |
| AxioCam MRm monochrome digital camera and Axiovision software | Zeiss | <https://www.zeiss.com/microscopy/en/products/cameras> |
| Cytation 3 cell imaging multi-mode reader | Biotek | <https://www.agilent.com/en/product/microplate-instrumentation/microplate-readers/multimode-microplate-readers/biotek-cytation-hybrid-multimode-reader-1623197> |
| Veriti 96-well thermal cycler | Applied Biosystems | <https://www.thermofisher.com/order/catalog/product/4375305> |

**References:**

1. Wilke G, Ravindran S, Funkhouser-Jones L, Barks J, Wang Q, VanDussen KL, et al. Monoclonal antibodies to intracellular stages of *Cryptosporidium parvum* define life cycle progression in vitro. mSphere. 2018;3(3):e00124-18. Epub 2018/06/01. doi: 10.1128/mSphere.00124-18. PubMed PMID: 29848759; PubMed Central PMCID: PMCPMC5976880.
2. Wilke G, Funkhouser-Jones L, Wang Y, Ravindran S, Wang Q, Beatty WL, et al. Forward genetics in *Cryptosporidium* enabled by complete in vitro development in stem cell-derived intestinal epithelium. Cell Host Microbe. 2019;doi.org/10.2139/ssrn.3331307.
