# Supplementary material for: Mendelian segregation and high recombination rates facilitate genetic analyses in Cryptosporidium parvum
Source: PLoS Genet. 2024 Jun 17;20(6):e1011162. doi: 10.1371/journal.pgen.1011162 (PMC11213348; doi:10.1371/journal.pgen.1011162)
Supplement: S2 Table — (DOCX) [file pgen.1011162.s007.docx]

**S2 Table.** Oligonucleotides and plasmids used in this study.

| **Usage** | **Full Plasmid name** | **Oligo name** | **Sequence (5’ – 3’)** | **Purpose** | **Source** |
| --- | --- | --- | --- | --- | --- |
| Epitope and fluorescent tagging of *cgd1_700* | pABC-3HA-CFP-Nluc-P2A-neo | ABCT Cter gibson-F | TGAATTCGAGCTCGGTACCCCTTCAATGGATATTGCCACAGATAA | Amplify CpABC1 C-terminus homology arm for building pABC-3HA-Nluc-P2A-neo by Gibson assembly | [1] |
|  |  | ABCT Cter gibson-R | GAGCCCGAGCCCTTGCTAGCAAGCTTATTAACTTCATTTGCAATTG |  | [1] |
|  |  | ABCT 3UTR gibson-F | TTTTTCTTTTTATCTTAATTTCCTCAAAACTGAGAGGATTTT | Amplify CpABC1 3'UTR homology arm for building pABC-3HA-Nluc-P2A-neo by Gibson assembly | [1] |
|  |  | ABCT 3UTR gibson-R | GTCGACTCTAGAGGATCCCCCGTGATTTTTATGACTCAAAAATTAT |  | [1] |
|  |  | ABC-PAM-F | AGTTGTACAATACTTTCAATTGCTCAC | Mutate PAM sequence of CpABC1 gRNA in pABC-3HA-Nluc-P2A-neo | [1] |
|  |  | ABC-PAM-R | AGAGAAATGTGTTTTGATAATATTCTGAA |  | [1] |
|  |  | Aldo 3UTR-F | ATGCATCTTCATTTAGTATCTTAGGT | Amplify pABC-3HA-Nluc-P2A-neo plasmid backbone for building pABC-3HA-CFP-Nluc-P2A-neo by Gibson assembly | [1] |
|  |  | 3HA-R | GGCATAATCTGGAACATCGTAAGG |  | [1] |
|  |  | pLinker-ECFP(gBlocks Gene Fragment) | ACGATGTTCCAGATTATGCCGCTAGCAAGGGCTCGGGCTCGACCCAGCTGATGGTGAGCAAGGGCGAGGAGCTGTTCACCGGGGTGGTGCCCATCCTGGTCGAGCTGGACGGCGACGTAAACGGCCACAAGTTCAGCGTGTCCGGCGAGGGCGAGGGCGATGCCACCTACGGCAAGCTGACCCTGAAGTTCATCTGCACCACCGGCAAGCTGCCCGTGCCCTGGCCCACCCTCGTGACCACCCTGACCTGGGGCGTGCAGTGCTTCAGCCGCTACCCCGACCACATGAAGCAGCACGACTTCTTCAAGTCCGCCATGCCCGAAGGCTACGTCCAGGAGCGCACCATCTTCTTCAAGGACGACGGCAACTACAAGACCCGCGCCGAGGTGAAGTTCGAGGGCGACACCCTGGTGAACCGCATCGAGCTGAAGGGCATCGACTTCAAGGAGGACGGCAACATCCTGGGGCACAAGCTGGAGTACAACTACATCAGCCACAACGTCTATATCACCGCCGACAAGCAGAAGAACGGCATCAAGGCCAACTTCAAGATCCGCCACAACATCGAGGACGGCAGCGTGCAGCTCGCCGACCACTACCAGCAGAACACCCCCATCGGCGACGGCCCCGTGCTGCTGCCCGACAACCACTACCTGAGCACCCAGTCCGCCCTGAGCAAAGACCCCAACGAGAAGCGCGATCACATGGTCCTGCTGGAGTTCGTGACCGCCGCCGGGATCACTCTCGGCATGGACGAGCTGTACAAGTGATTAATATGCATCTTCAT | Building pABC-3HA-CFP-Nluc-P2A-neo plasmid by Gibson assembly | This study; IDT |
|  | pACT:Cas9, U6:sgABC | ABC gRNA-linker | CTGAAAGGTTAAGTGTTGGGGAATTCTGTGAGCAATTGAAGTTTTAGAGCTAGAAATAGC | Building Cas9 plasmid for CpABC1 tagging by Gibson assembly | [1] |
|  | Confirming insertion | ABC-F1 | gcaagagccatacttagaaagagtaag | To check correct gene insertion from DNA purified from mouse feces. | [1] |
|  |  | pEno R | tagagtttggtgtgcaggcg |  | [2] |
|  |  | Neo F1 | GCTGAAGAACTTGGTGGTGA |  | [2] |
|  |  | ABC-R1 | ctccttagtatggactttgggg |  | [1] |
| Epitope tagging of *cgd5_1960* | Enolase-3HA-Nluc-P2A-neo | Enolase 5'UTR Gib F1 | TGAATTCGAGCTCGGTACCCATTGACAAGCCAAGTTGGTG | Amplify CpEnolase C-terminus homology arm for building Enolase-3HA-Nluc-P2A-neo by Gibson assembly | This study; IDT |
|  |  | Enolase 5'UTR Gib R2 | GAGCCCGAGCCCTTGCTAGCTTATCTGATTCTTGCGATGGGTT |  | This study; IDT |
|  |  | Enolase Gib 3'UTR F2 | ACGAATTCTTCTGACAATTGGTTCGTGGCGTGTAGGATAG | Amplify CpABC1 3'UTR homology arm for building pABC-3HA-Nluc-P2A-neo by Gibson assembly | This study; IDT |
|  |  | Enolase Gib 3'UTR R2 | GTCGACTCTAGAGGATCCCCTTGGGGCACATTATTCAGACT |  | This study; IDT |
|  |  | Enolase PAM MUT gRNA2 F2 | AACTGGCGCGTCTTGTAGAAGTGA | Mutate PAM sequence of *Cp*Enolase gRNA in Enolase-3HA-Nluc-P2A-neo | This study; IDT |
|  |  | Enolase PAM MUT gRNA2 R | TTAAGTTGGCCTGTACCTAGGCCAAC |  | This study; IDT |
|  | pACT:Cas9, U6:sgEnolase | Enolase gRNA2 Linker | CTGAAAGGTTAAGTGTTGGGGCAACGCGCTCACTTCTACAGTTTTAGAGCTAGAAATAGC | Building Cas9 plasmid for CpEnolase tagging by Gibson assembly | This study; IDT |
|  | Confirming insertion | Enolase insert F1 | CATATGCTTCATTGACAAGCCAAGT | To check correct gene insertion from DNA purified from mouse feces. | This study; IDT |
|  |  | pEno R | tagagtttggtgtgcaggcg |  | [2] |
|  |  | Neo F1 | GCTGAAGAACTTGGTGGTGA |  | [2] |
|  |  | Enolase insert R1 | TTAACATTAATGAGATTGGGGCACATTATT |  | This study; IDT |

**References:**

1. Xu R, Beatty WL, Greigert V, Witola WH, Sibley LD. Multiple pathways for glucose phosphate transport and utilization support growth of Cryptosporidium parvum. 2024. doi: 10.1038/s41467-024-44696-3. PubMed Central PMCID: PMCPMC1032708.
2. Xu R, Feng Y, Xiao L, Sibley LD. Insulinase-like Protease 1 Contributes to Macrogamont Formation in Cryptosporidium parvum. mBio. 2021;12(2). Epub 2021/03/11. doi: 10.1128/mBio.03405-20. PubMed PMID: 33688009; PubMed Central PMCID: PMCPMC8092296.
