# Supplementary material for: Mendelian segregation and high recombination rates facilitate genetic analyses in Cryptosporidium parvum
Source: PLoS Genet. 2024 Jun 17;20(6):e1011162. doi: 10.1371/journal.pgen.1011162 (PMC11213348; doi:10.1371/journal.pgen.1011162)
Supplement: Attachment — Submitted filename: Resposne to reviewers.docx [file pgen.1011162.s009.docx]

Reviewer's Responses to Questions

**Comments to the Authors:
Please note here if the review is uploaded as an attachment.**

**Reviewer #1:**

One minor point; Could the authors clarify the analysis for quantitating oocyst shedding in mouse experiments which is based on a single sporozoite giving rise to an oocyst (line 400 - the average oocyst count is divided by four, since the oocyst contains four sporozoites). The calculation does not seem to take into account that not all merozoites (the progeny of sporozoites) result in male and female gametes. This may explain why they do not see perfect outcrossing from in vivo experiments.

**Response**:

Thank you for your thoughtful comment. The qPCR analysis described in the text (line 400) was only used for monitoring the amplification of transgenic parasite lines prior to oocyst purification and subsequent use in crossing experiments. qPCR was not used for the quantification of outcrossing or selfing in *in vivo* experiments.

**Reviewer #2:**
Major comment: Perhaps I have misunderstood something, but in the cross in Figure 2, I don’t understand why the authors expect the frequency of Tetratype (TT) oocysts to be low. This cross involves markers that are on two different chromosomes (uprt-mCherry on chr. 1, and tk-GFP on chr. 5) so they should segregate independently, as the authors indeed say on P7 L178. So I don’t understand why the authors then go on to say that the TT class is only possible “if crossover occurs during interphase I near the uprt or tk locus” (L184), and they don’t cite any reference to support this statement. They then observe that 87-89% of the oocysts are TT (Fig. 2B,D) and they say on line 200 that this frequency is surprisingly high (also on line 229).
In yeast genetics, a cross involving two markers on different chromosomes would result in the ratio of asci classes being 1 PD : 1 NPD : 4 TT, (i.e. 66% of the asci will be tetratype), unless each of the markers is tightly linked to the centromere of its chromosome. See Hiten Madhani’s online notes about yeast tetrad analysis, <https://studylib.net/doc/8744127/classical-genetics--tetrad-analysis-and>
The manuscript doesn’t say anywhere that the UPRT and TK genes are centromere-linked, so I presume that they are not. So shouldn’t the question be whether the observed 87-89% frequency is statistically higher than the expected 66% ?

**Response:**

Thank you for pointing this out as the source has been quite helpful in helping us think about the outcome of our crosses. The summary provided above (1:1:4) would apply for markers distal from centromeres were recombination is high. The same source cited also indicates the ratio is 1:1:1 for markers that are proximal to centromeres or were recombination rates are low. In Cp we do not know where centromeres are although we are investigating this point in separate studies. When we first designed Cp crosses, we were influenced by our prior work with *Toxoplasma gondii*. In that system, crosses between independent markers (various drug resistant markers) were used to test the outcomes of meiosis in bulk segregation assays first conducted by Elmer Pfefferkorn in the 1980s. Such crosses lead to a 50% outcrossing and 50% selfing rations since single, cloned haploid parasites can make either gamete (something also verified for Cp). The frequency of doubly drug resistant mutants in such crosses was typically around 12.5% as predicted for two unlinked markers on separate chromosomes that each undergoes only low levels of crossover. Subsequent linkage mapping studies in Tg also demonstrated relatively low recombination rates, which would produce rare TT genotypes among unlinked markers on separate chromosomes. Our initial assumption was that these parameters might be similar in Cp, so were surprised by the high frequency of TT. We did not appreciate that this would be the predicted outcome for markers distal from centromeres with a high recombination rate- as suggested for the example above for yeast. However, there are other possible outcomes that could drive high TT outcomes, even from markers with intrinsically low recombination, such as inverted meiosis. We have revised the text to point out these different outcomes and to contrast the behavior of Cp chromosomes in light of possible different models. Importantly, the subsequent single chromosome analyses clearly distinguish among these models- and support the high crossover frequency and ratio of 1:1:4 as suggested above.

**Minor comments:**

Lines 36-37: As a non-specialist, I did not know what an oocyst is until I read the Introduction, so I didn’t initially understand these lines in the Abstract. It would be helpful if you could modify line 36 to say something like “Infection of ALI cultures or Ifngr1 mice with mCherry and GFP parasites resulted in cross-fertilization and formation of “yellow” oocysts (clusters of 4 haploid cells segregating from a meiosis)”.

Response: Revised as suggested.

Lines 120-122 make analogies to classical genetic analysis in yeast, referring to “random oocyst analysis” and “oocyst tetrad dissection”. It would be nice to return to these analogies in the Discussion to discuss them in more detail. The experiment in Figure 1 is random oocyst analysis which in yeast would be equivalent to random ascus analysis (not random spore analysis). The experiment in Figure 2 is oocyst tetrad dissection, OK. The experiments in Figures 3 and 4 are, I think, equivalent to random spore analysis.

Response: Thank you for making this excellent suggestion, such terminology is not commonly used in the parasite field but would be appropriate and a welcome addition. We agree that that Figure 1B is equivalent of a random ascus analysis, but 1D, 1E and figures 3 and 4 are random spore analysis. We have revised as suggested.

Line 183, “parental non-ditype” should be “non-parental ditype”.

Response: Corrected.

In Table 1, please add two columns showing “% of TT oocysts” and “% of TT + other oocysts”.

Response: Revised as suggested.

P11 L275, spell out Cp as “C. parvum”.

Response: Revised as suggested.

Reviewer #3: In this paper, isogenic lines of Cryptosporidium parvum were fluorescently tagged with mCherry or GFP on chromosomes 1 or 5 and allowed to undergo hybridization to investigate the frequency of crossing over, and whether chromosomes segregate independently in a Mendelian manner. To do this, the authors took advantage of a method they developed to culture all stages of Cryptosporidium in stem cell derived intestinal epithelial cells grown under air-liquid interface conditions (ALI), which they published in Cell Host and Microbe in 2019. Growth in ALI cultures generates infectious oocysts that are “yellow”, products of cross-fertilization between a red and green parental line. By FACS purifying “yellow” oocysts, and plating these oocysts in conditions that allowed for individual sporozoites from each oocyst to be counted, a technique the authors dubbed as “oocyst tetrad dissection”, the phenotypes of each individual sporozoite from single oocysts were quantified. The authors also produced additional lines that possessed tags that were linked (on the same chromosome), to calculate crossover frequencies, and to determine a recombination rate for each of the two chromosomes queried, which they estimated at between 3-12.1 kb/cM. This work is significant because the researchers applied new assays to study meiosis in Cryptosporidium and they showed, by examining progeny from crosses between transgenic parasites, that Cryptosporidium utilizes both self-fertilization and cross-fertilization, has high rates of recombination, and that chromosomes segregate in a Mendelian manner. In an effort to compare / contrast their results with those of fungi, in which tetrad analysis is pursued as a genetic technique to study recombination events and meiosis, the researchers used terminology such as parental ditype (PD), non-parental ditype (NPD) and tetratype (TT), and specifically showed that yellow oocysts, generated by fertilization between a gamete that had the red fluorescent tag with a gamete that had the green fluorescent tag, largely produced (ranging from 85-89%) an equal frequency (or 1:1:1:1 tetratypes) of progeny that were either dark, red, green, or yellow. This was expected. However, the researchers found this to be unexpected (see Fig 2A), which appears to be a flaw in their logic and needs to be corrected, or better explained.

Response: We did not find the frequency of overall genotypes to be surprising, indeed they match what would be expected for segregation of two unlinked markers in a cross among haploid parental strain. What we found surprising, is the high frequency of TT that occurs from a single oocyst, which suggests that crossover occurs with very high frequency. Please see the explanation above for Reviewer #2.

Major Points:
1. Figures – are generally well received, and the graphics are excellent summaries of the data, but are lacking the visual display of the raw data. In referring back to the CHM paper in 2019, the figures in that manuscript were admixed with raw data plus summarized results. I am not necessarily asking for the same here, but more extensive experimental data could be added to the supplemental section, as seen for example in Suppl. Fig. 1, which provided real insight into some of the nuances of the dataset, and how timing is critical for data collection, to avoid the complications associated with a second round of meiosis, or the time needed post meiotic reduction (14hpi) to sufficiently dilute the inherited cytosolic fluorescent protein of the other parent, to establish which germline encoded fluorescent protein remained stably expressed for a more precise tabulation in the results.

Thank you for these suggestions. We have generated a new Figure S2 analyzing the time of parentally inherited fluorescent protein degradation and the time of germline encoded fluorescent protein expression over six different fields of view. We have also provided a new file (S1 Dataset) which has the raw data and summarized results for Figures 1B, 1D, 1E, 2B, 2D, 3C, 4C, S2 and the calculations used to estimate the rate of recombination.

2. Figure 2A – the logic appears to be flawed, and needs to be better explained, and/or corrected. Cryptosporidium is haploid, no genome reduction occurs to produce gametes, rather a developmental queue commits each haploid parasite to differentiate into either a microgamete or a macrogamete, and upon fusion, a 2n zygote forms. There are no perceived mating types, and from the analyses in this paper, there appears to be an equal frequency of self- vs. cross-fertilization occurring. Hence, there is a 50% chance of being either red or green, or 50% chance of being yellow for the two unlinked markers. And for the oocyst tetrad dissection analysis, the predicted outcome would be a 1:1:1:1 ratio in this scenario, hence dark:red:green:yellow progeny (or TT) from each single, yellow oocyst. This is largely what has occurred (Fig 2B and Fig 2D), and is what is predicted, so it is unclear why Figure 2A depicts the opposite, and a paragraph of text is devoted to this as an unexpected result. Minor deviations from this were evident in the results shown. It was the same whether oocyst formation was in vitro in ALI culture conditions, or in vivo in immune-suppressed mice, but that the non-TT frequency was minimal, and was less than 10% - distributed as PD, NPD, or other. As described above, some of the complications of the experimental conditions (whether a second round of meiosis had begun, or dilution of the non-germline encoded marker obscured truth) could have accounted for this deviation. I believe this section of the results and discussion should be re-considered, and revised with the knowledge disclosed above. It may prove advantageous to generate a pictorial schematic for the segregation of two genes in a cross, that are unlinked, and also not linked to the centromere of the chromosome they are encoded on.

Response: Please see the response to a similar question by Reviewer #2.

3. Figure 1B. The over-representation of the Green oocysts in the red x green cross between isogenic lines transgenic for the two fluorescent markers is intriguing. The authors suggest that this was the result of a greater frequency of selfing by the GFP tagged strain. Do we know if this is indeed the case? Is this a numbers effect? Does the GFP strain out-proliferate the mCherry strain? If you were to perform qPCR at various times post inoculation, do you in fact see an increase in the GFP strain, at the expense of the mCherry strain to confirm that it expands more quickly. If indeed that is the case (that it is not some intrinsic difference between the two transgenic clones), then an experiment that adds an unequal amount of say 2x mCherry sporozoites to 1X GFP sporozoites (or some empirical variation on this) is warranted, and would that generate the expected 50% yellow, 25% red and 25% green?

Response: We have not conducted a careful quantitative analysis of the growth rates of these strains in vivo as this was not the goal of our study. Although the analysis suggested could provide interesting insight, we do not think it would be beneficial to add it to the current manuscript. Instead, we are offering the suggestion that the GFP expressing strain is better at selfing as a plausible explanation for the skewed ratios. We have added a caveat statement, that this could also reflect some unknown fitness difference as a result of generating the transgenic strains.

4. Figure legends and text throughout. The term “F1” was applied whether it was for “selfing” or for “cross-fertilization” between the two strains that were tagged with different colors. Sometimes that led to confusion. For example, when the F1 label was applied to a yellow oocyst graphic, but the text read Progeny of F1 oocysts and discussed self as well as cross-fertilization. Is it possible to change throughout the legends and text accordingly: “progeny of ‘yellow’ F1 oocysts” or ‘red’ F1 oocysts– to infer that the analysis was done on oocysts derived from a mating experiment, but that the specific analysis pursued was performed on F1 oocysts of a specific color type.

Response: Revised as suggested.

5. Do we know where the centromeres are in Cryptosporidium? I did not look, but I presume the answer is no. But if we do, knowing that information, and how close the markers are to the centromeres would be important to add into the text.

Response: We have added a statement to the text to indicate this information is not known and tied it to the discussion of different models for recombination.

Minor Points:
1. Why are there two different abstracts? The one pasted into the PLoS Genetics cover page is different from the one in the Word file associated with the document. Please synchronize abstracts

Response: Corrected.

2. Page numbering was not included in the files received for review. On page 3, second paragraph, line 3 – change “oocysts” plural to “oocyst”

Response: Corrected.

3. Page 4, tenth line, change “…has enabled the discovery of virulence factors…”]

Response: Corrected.

4. I am struggling with the term “outcrossed” – page 5, line 7, and throughout the Discussion. These are isogenic lines, that are tagged transgenically with fluorescent markers. In effect, is it more appropriate to refer to these as unisexual crosses?

Response: We can appreciate the concern but are not convinced that “unisexual cross” would be appropriate since any cross among apicomplexan parasites requires both male and female sexes, although they can emanant from one cloned cell. More appropriately, we have adopted “single lineage crosses using isogenically tagged strains”, to distinguish selfing from crossing.

Or some phrase that describes their similarity in genetic origin, since other genetic factors may influence the outcome in outcross mating experiments between strains that are more highly polymorphic, and have different origin / ancestry.

Response: We agree that crosses between different parental types may be greatly influenced by difference in genes, and indeed such crosses are underway and appearing on other papers currently. We have added a statement to the discussion to highlight these important differences.
